# Optimized Classifier Learning for Face Recognition Performance Boost in Security and Surveillance Applications

**DOI:** 10.3390/s23157012

**Published:** 2023-08-07

**Authors:** Jitka Poměnková, Tobiáš Malach

**Affiliations:** 1 Department of Radio Electronics, Faculty of Electrical Engineering and Communication, Brno University of Technology, Technicka 3082/12, 61600 Brno, Czech Republic; 2 EBIS, spol. s r.o., Krizikova 2962/70a, 61200 Brno, Czech Republic

**Keywords:** template creation, surveillance face recognition, classifier learning, parameter optimization, security application

## Abstract

Face recognition has become an integral part of modern security processes. This paper introduces an optimization approach for the quantile interval method (QIM), a promising classifier learning technique used in face recognition to create face templates and improve recognition accuracy. Our research offers a three-fold contribution to the field. Firstly, (i) we strengthened the evidence that QIM outperforms other contemporary template creation approaches. For this reason, we investigate seven template creation methods, which include four cluster description-based methods and three estimation-based methods. Further, (ii) we extended testing; we use a nearly four times larger database compared to the previous study, which includes a new set, and we report the recognition performance on this extended database. Additionally, we distinguish between open- and closed-set identification. Thirdly, (iii) we perform an evaluation of the cluster estimation-based method (specifically QIM) with an in-depth analysis of its parameter setup in order to make its implementation feasible. We provide instructions and recommendations for the correct parameter setup. Our research confirms that QIM’s application in template creation improves recognition performance. In the case of automatic application and optimization of QIM parameters, improvement recognition is about 4–10% depending on the dataset. In the case of a too general dataset, QIM also provides an improvement, but the incorporation of QIM into an automated algorithm is not possible, since QIM, in this case, requires manual setting of optimal parameters. This research contributes to the advancement of secure and accurate face recognition systems, paving the way for its adoption in various security applications.

## 1. Introduction

Safety, security, and the mitigation of security risks have assumed importance in contemporary times. A low level of security can increase the risk of events that can have serious consequences for society, the economy, or the environment. As a result, security standards are being consistently augmented across various domains, encompassing critical infrastructure, public transport, government buildings, sports arenas, and social venues. Traditional security solutions, such as intrusion detection, access control, and surveillance closed circuit television (CCTV) systems, are being enhanced by new technologies, such as biometric identification. Fingerprint or hand geometry identification systems have been used for several years, and systems based on palm vein or iris scans are currently emerging.

This paper presents an enhanced face recognition technology tailored for edge computing devices, with a primary focus on CCTV cameras utilized in security applications. Requirements for enhancing security grow in various facilities and applications. Face recognition is no longer confined solely to highly secure environments such as critical infrastructure or banking institutions. It has gradually spread to many other industries, for example, border security, marketing and retail applications, and construction sites.

The utilization of standalone face recognition systems is gradually diminishing, and there is a growing demand for integrating these systems with other complementary solutions. In particular, facility security applications require its integration with access control, time and attendance, and others in order to build a complex security solution that fulfills functional and administrative requirements. The integration with other systems allows the addition of more clues into face recognition algorithms, which permits, firstly, face recognition operation in edge computing or Internet of Things (IoT) devices and, secondly, an increasingly correct face recognition rate. Both can be achieved by intelligent management of a template database. The management of a template database enables us to add, delete, activate, or deactivate a face template, which, according to information from other systems, can instantly optimize a number of templates, bringing the above-mentioned benefits.

Deep learning of neural networks has emerged as another promising approach for general face recognition, gaining significant support both at the software and hardware level [1,2,3]. However, face recognition has not been applied yet due to computational power on edge devices; usually, pre-processing methods such as face detection are applied [4,5]. The solution we propose is to manage a template database to reduce the requirements for computational accuracy. Neural networks utilize a classifier learning process, which requires learning the classifier with all possible training data, resulting in the learned network being able to classify trained data. The request to manage database templates of different training data requires relearning the network, which is not feasible due to the required time and computational demands. Therefore, we experiment with the nearest neighbor classifier, which allows instant changes in face templates and template database management. In summary, while deep learning of neural networks shows great promise, it may not be suitable for our specific application. Instead, our approach, centered on template database management and the utilization of a nearest neighbor classifier, offers a viable alternative to meet our computational requirements while maintaining recognition performance.

In the industry, there is a growing interest in shifting computing tasks from servers to edge devices. However, video analytics, especially when applied to multiple cameras, pose a challenge in terms of computational power. Thus, cameras with a higher computational power aim to run video analytics. Some camera vendors provide open platforms for third party applications to run on their cameras, e.g., the Axis camera application platform [6]. Our face recognition system is intended for such an implementation as it could be operated solely by a camera including the whole face recognition process, i.e., face detection, face description, and classification. The proposed face recognition system is designed to allow easy and rapid face database management based on information from other systems. It enables face recognition in more frames, which enhances the correct recognition rate and user experience (e.g., gates or barriers at which the person is not allowed to enter until recognition), and the time required for recognition is minimized.

The research presented in this paper builds upon previously published findings in the field of face recognition for security applications. The face recognition system employed in this study incorporates the Viola–Jones face detector, local binary pattern histograms (LBPH) for face description, and a nearest neighbor classifier. The local binary patterns were also used in other parts of computer vision, such as detection [7], classification [8], description of interest regions [9], or matching [10]. The nearest neighbor classifier is utilized because it facilitates rapid management of a template database, which is essential for applications in security cameras as further described. The central focus of this research lies in the creation of a template for the classifier. As documented in our work [11], a suitable representation of a face contributes to the improvement of the face recognition rate. Therefore, we investigate a template creation process to enhance the correct recognition rate, and we use the face recognition systems described above as they are convenient for implementation in security systems and edge computing devices. We discuss this issue further in the following section.

### Paper Contribution and Organization

Various methods for template creation have been introduced in the literature [11,12,13]. The main objective of this paper is to offer a comprehensive performance comparison of template creation methods while conducting an in-depth study of selected methods and their characteristics. To achieve this, we have included seven template creation methods for comparative analysis. In this regard, we provide a brief introduction to four commonly used traditional template creation methods and three state-of-the-art methods. These methods are categorized into two groups based on their nature: cluster description-based methods and cluster estimation-based methods.

Most results were evaluated on images from the IFaViD database [14], which comprises real-world scenarios and applications captured by security cameras. In our prior work, we used IFaViD database images captured in two scenarios represented by datasets A1 and B (for a detailed description see [11]). In this study, we used a database extended by the dataset A2, which consists of 62,801 images and extends the overall number of images in the database nearly four times (see Table 2). Additionally, we conduct a new analysis that distinguishes between open- and closed-set identification. The database and the testing methodology respect the operational evaluation presented by Phillips et al. [15], i.e., they assess the recognition performance for a specific algorithm in a particular application context. Consequently, the presented results are more representative of the intended application and support the general idea that template creation is worthy of investigation since the optimal face template creation can improve recognition.

In our previous research, we found out that the quantile interval method (QIM) method is a promising method for creating a face template in the face recognition process [11]. Previously, QIM was solely compared with the centroid method, while the presented paper proposes a comparison with other above-mentioned methods. Moreover, while the previous article employed a simple setting of quantiles based on statistical outlier detection, the present paper now pays closer attention to the assessment and optimization of quantiles, as illustrated by Figures 11 and 12. Thus, based on the presented work, we are able to define a methodology and procedure for setting the parameters of the quantile interval method. Setting parameters is substantial for the practical application of template creation methods.

The presented paper offers three contributions: (i) We strengthened the evidence that the quantile interval method outperforms the contemporary approaches. Furthermore, (ii) we extend the database of images captured by a real camera system containing complex scenarios and propose more comprehensive testing performed on this database. We used two scenarios representing surveillance applications and one scenario of an access control application. In addition, we distinguish between an open- and closed-set identification. Moreover, (iii) we perform an evaluation of the cluster estimation-based method (QIM) with an in-depth analysis of the quantile setup for the test set of the scenario representing surveillance applications. We provide instructions and recommendations for the correct identification of parameter setup.

The organization of the paper is as follows: First, an introduction is presented. The methodology is explained in Section 2. Then, the IFaViD (IVECS Face Video Database) dataset, scenarios, and evaluation metrics are thoroughly described in the Section 3. In Section 4, an application to the data is made. Finally, the results are presented in Section 5, and the conclusion is presented in Section 6.

## 2. Methodological Overview

**Figure 1:**
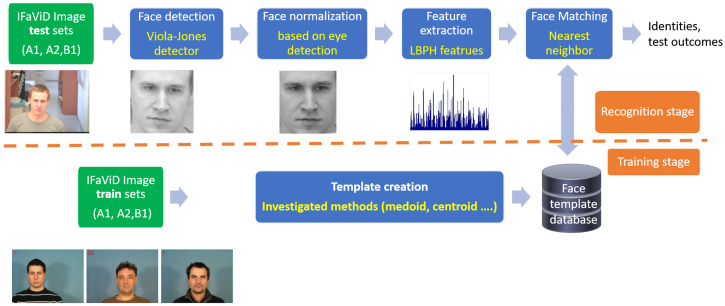
Face recogniton system. Particular algorithms are highlighted with yellow color.

In this section, we introduce the face recognition system, consisting of two key stages: the recognition stage and the training stage. We elaborate on the recognition stage and its modules. Additionally, we emphasize the significance of the template creation (TC), also referred to as the classifier learning process, for the effective functioning of the recognition stage. Template creation is one of several algorithms that constitute the face recognition (FR) system depicted in Figure 1. TC methods target processing training data in order to provide such a representation of the data that would enhance the recognition performance, and, thus, they are closely related to cluster analysis.

The FR system includes four basic modules in the recognition stage, i.e., a face detection module, face normalization, a feature extraction module, and a face-matching module.

Localization of a face in the image is performed via a Viola–Jones face detector [16] in the first face detection module. Face normalization includes face alignment and scaling. It is based on the detection of eyes. The face image is rotated using an affine transform so that the eyes are horizontally aligned. The face scale is determined according to the inter-eye distance. In the feature extraction module, the detected face is characterized by a local binary pattern (LBP) histogram features [17,18]. LBPH features are extracted by the application of an LBP operator to a face image. The LBP operator enhances and captures the edges of facial features. The image is subsequently divided into non-overlapping regions. A histogram of responses to the LBP operator is computed separately in every region. The histograms are then concatenated and constitute the LBPH feature vector. For later use, the histogram can be characterized by a suitable probability density function (PDF). In the face-matching module, features representing a face are classified. Here, we apply the nearest neighbor (NN) classifier [17,19]. The relationship between a feature vector and face templates is assessed using a $\chi^{2}$ dissimilarity metric. Dissimilarity is computed among the feature vectors and all face templates. Then, the feature vector is assigned to a face template whose mutual dissimilarity is the smallest among all, and the dissimilarity does not exceed defined thresholds.

The described system is used for the evaluation of template creation methods. The created templates serve as reference patterns for each individual. In the following two sections, we specifically focus on template creation and present a detailed exploration of these methods based on their nature (template description methods and template estimation methods). Under template description methods, we explore the medoid method, advanced medoid method, centroid method, and weighted centroid method. The cluster estimation-based methods consist of the Gaussian mixture model method, QIM, and HQM. In this study, in line with the stated objective, we evaluate each method’s influence on the recognition performance of the system described earlier, utilizing the extended database. The cluster description-based methods (namely centroid, weighted centroid, and medoid) and Gaussian mixture model method were also used in our previous research [11,20].

### 2.1. Template Creation Methods Based on Cluster Description

Template creation methods characterize clusters of training feature vectors using descriptive statistical measures. These TC methods apply statistic descriptors directly to training data.

#### 2.1.1. Medoid Method and Advanced Medoid Method

The medoid method (Medoid) is a traditional approach to TC. A medoid is a member object of a training set whose dissimilarity to all other objects in the training set is minimal [21]. In terms of face TC, the medoid method selects the training feature vector whose accumulated dissimilarity is minimal as a template. The accumulated dissimilarity is computed based on the $\chi^{2}$ distance metric defined as:

(1) $$\chi^{2}\left(P,Q\right)=\sum_{i=1}^{N}\frac{{\left(P_{i}-Q_{i}\right)}^{2}}{P_{i}+Q_{i}},$$

where *P* and *Q* are feature vectors, and *N* is the dimensionality of the feature vectors. The medoid method produces face templates that represent real faces (e.g., Prinosil in [22]). We propose a TC method called the advanced medoid method (Advanced medoid) which is based on the medoid method and includes an outlier removal feature. The outlier removal feature aims to remove training feature vectors that are not representative and, therefore, not suitable for template creation.

The advanced medoid method uses the $\chi^{2}$ distance (Equation (1)) between all training feature vectors of a single individual and performs outlier removal by thresholding the distances. The training feature vectors with accumulated $\chi^{2}$ distances that exceed the defined threshold th are excluded. The $\chi^{2}$ distances among the remaining feature vectors are scaled using the following formula:

(2) $$D^{\prime }\left(P,Q\right)=\sqrt{\frac{th-\chi^{2}\left(P,Q\right)}{th}}.$$

Each feature vector is assigned a sum of scaled distances $D^{\prime }\left(P,Q\right)$. The resulting face template is created from those feature vectors that have the highest sum value.

#### 2.1.2. Centroid Method and Weighted Centroid Method

The face template produced by the centroid method (Centroid) is not limited to the existing training feature vectors that represent a real face. The centroid method represents a cluster, formed by training feature vectors, by the cluster centroid. This method expects that all vectors have the same significance. The ultimate face template is computed as a mean vector in all dimensions. The face template $T_{i,j}$ for an individual *i* in the dimension *j* is determined as follows:

(3) $$T_{i,j}=n_{i}^{-1}\sum_{i=1}^{N}f_{i,j,n},$$

where $f_{i,j,n}$ is the value of the *n*-th feature vector, $n_{i}$ is the total number of feature vectors, both corresponding to the i,j-th individual, resp. dimension.

We propose the optimization of the centroid method, called the weighted centroid method (Weighted centroid). This method limits the contribution of selected feature vectors in the process of weighted centroid computation. This limitation reflects the need to cope with possibly unsuitable and outlying training feature vectors.

A strict rejection of some training features, used in the advanced medoid method, does not appear optimal. Therefore, the weighted centroid template computation is based on weighting the feature vectors, which exceed a certain dissimilarity level in the process of centroid calculation. The weighting is represented by the limitation of feature vector contribution. The weighted centroid template $Tw_{i,j}$ for the individual *i* in the *j*-th dimension is computed as follows:

(4) $$Tw_{i,j}=T_{i,j}+w_{i,j}\left(f_{i,j,n}-T_{i,j}\right),$$

where $T_{i,j}$ is the cluster centroid (computed according to Equation (3)) for the individual *i* in the *j*-th dimension, $w_{i,n}$ is the weight of the *n*-th feature vector of the individual *i*, and $f_{i,j,n}$ is the value of the *n*-th feature vector of the *i*-th individual in the *j*-th dimension. Then, the distances of the training feature vectors from the respective centroid are computed. Distant features are assigned a lower weight, and their contribution is thus limited, bringing them closer to the centroid. These distant features can cause a deflection from the centroid’s optimal position. The weighted centroid method should provide a better representative point of a cluster, resulting in a higher recognition performance [11].

### 2.2. Template Creation Methods Based on Cluster Estimation

Cluster estimation-based methods for TC estimate the distribution of training feature vectors. Then, various methods to characterize the distribution resulting in a construction of a template are applied. These methods describe the estimated distribution of data rather than the data themselves, which is an essential difference from the methods based on cluster description.

#### 2.2.1. Gaussian Mixture Model-Based Method

The distribution of the training feature vectors is discovered by utilizing the expectation–maximization (EM) algorithm. The EM algorithm estimates a Gaussian mixture model (GMM) describing the distribution of the training feature vectors [12]. The face template is determined as a weighted sum of the Gaussian components’ means.

The GMM is estimated by the EM algorithm in each dimension of the feature vector separately. The one-dimensional GMM p(x) is defined as $p\left(x\right)=\sum_{k=1}^{K}w_{k}N\left(\mu_{k},\Sigma_{k}\right),$ where $w_{k}$ are the weights of generally multivariate normal distributions determined by the vector of the means $\mu_{k}$ and the covariance matrix $\Sigma_{k}$, and *k* is the number of Gaussian components in the GMM. GMMs are estimated in all dimensions of the training feature vectors. The GMM-based face template $T_{GMM(i,j)}$ is computed as a weighted sum of the means as follows:

(5) $$T_{GMM(i,j)}=\sum_{k=1}^{K}w_{i,j,k}\mu_{i,j,k},$$

where $w_{i,j,k}$ is the weight of *k*-th component in *j*-th dimension of the feature vector for the *i*-th individual, and $\mu_{i,j,k}$ is the mean of the normal distribution representing the Gaussian component. The number of components *K* is determined separately for each dimension (feature) according to the quality of the GMM fit, which is expressed by the Akaike information criterion [23]; the possible number of components *K* is in the range from 1 to 15.

#### 2.2.2. Quantile Interval Method

We conducted an analysis of dissimilarities among templates and test feature vectors. It revealed that the dissimilarity of the template and well-aligned, well-illuminated face with a neutral expression and face pose is not negligible. This led us to the construction of a template that would be tolerant of certain dissimilarities which are natural. Therefore, we propose the quantile interval method (QIM) [11].

The main idea of QIM is based on the fact that we allow a small variability of faces limited by thresholds forming a tolerance interval in which the degree of dissimilarity between the template and the feature vector is considered to be zero. The resulting face template is thus formed by combining the templates of all features, where the template for one feature is created with respect to the quantiles specified. The templates thus represent a multidimensional feature cluster determined by space.

To avoid the effect of the variability of one person’s feature on the overall dissimilarity of the templates, we use a modified $\chi_{M}^{2}$ distance when creating the tolerance interval templates and calculating the dissimilarity between the feature vector and the face template

(6) $$\chi_{M}^{2}\left(H,T\right)=\sum_{i=1}^{N}\left\{\begin{array}{cc} \;\frac{{\left(th_{2,i}-H_{i}\right)}^{2}}{th_{2,i}+H_{i}} & th_{2,i}<H_{i}\\ \;0 & th_{2,i}<H_{i}<th_{1,i}\\ \;\frac{{\left(th_{1,i}-H_{i}\right)}^{2}}{th_{1,i}+H_{i}} & th_{1,i}>H_{i} \end{array}\right\},$$

$th_{1,i},th_{2,i}$ are thresholds of the face template *T*, *H* is the feature vector of the unknown face, and *N* is the number of features.

**Figure 2:**
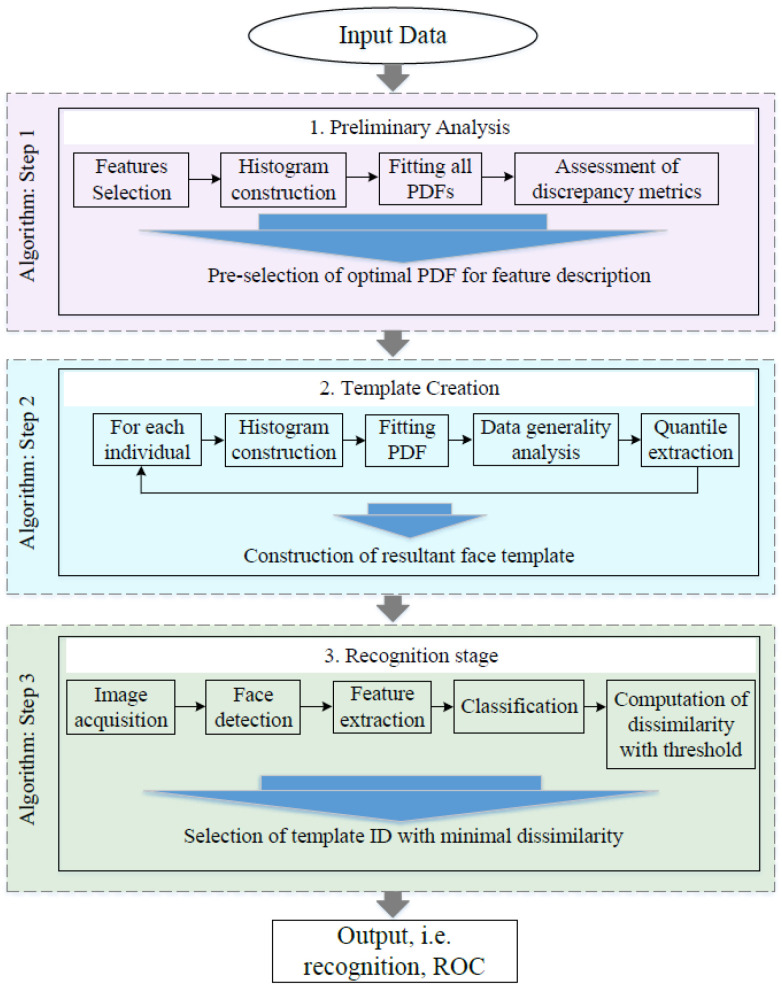
Three-step algorithm of template creation and face recognition.

The methodology of the template creation and face recognition is shown in the diagram (Figure 2).

#### 2.2.3. Higher Quantile Method

The resultant template created by QIM is twice as long as the template computed by the other presented methods. This is considered to be a disadvantage that may result in higher memory or computational power requirements. Reducing the computational complexity of the QIM can be achieved in some cases by using only a higher quantile to determine the template, as shown by an additional analysis of the QIM tolerance intervals. This modification is therefore called the higher quantile method (HQM).

In some cases, the lower thresholds can be omitted without affecting the template properties and the resulting recognition. This can be performed in a situation in which the lower thresholds for the PDF are close to each other and close to zero. Then, the HQM method works only with the upper threshold, resulting in a shorter template length compared to the length of the template created by the QIM and in a faster computation.

## 3. Data

The influence of the TC method on the FR system performance is evaluated on the IFaViD (IVECS Face Video) database. IFaViD is a database of video sequences and images captured by a model surveillance camera system and access control system. It is the representation of a real-world camera system by a database of video sequences and images. The model camera system was used for video sequence capturing which was assembled into a database. The IFaViD database aims to capture all mentioned negative influences that may occur in real-world operation.

In the case of surveillance face recognition, general databases (e.g., FERET [24] or LFW [25]) would not provide credible results. Therefore, similar to [22] or [26], we used a specific dataset to examine the performance of a selected application.

The data for database assembly were captured by the model camera system, which had been designed to enable collecting video sequences that would trustworthily represent surveillance CCTV and an access control system. The model camera system was designed to represent two scenarios: (i) A—a dynamic scenario (walking person), which represents surveillance applications; (ii) B—a static scenario, which represents a typical access control application.

### 3.1. IFaViD Database Assembly

A model camera system using three cameras was built. There were two cameras for scenario A and one camera for scenario B. The video sequences were captured for a period of half a year. The video sequence capturing was controlled by motion detection. The captured video sequences were manually sorted, i.e., video sequences with a person’s back that did not contain any face were discarded, and video sequences of very low quality due to extreme illumination conditions or face occlusion (big sunglasses, hats, caps, etc.) were also discarded.

The database contains three sets named according to the particular scenario and camera site. These are sets A1, A2, and B1. Each set has its own test set and training set. In this paper, we utilized set A2 for the first time, and we newly present recognition performance on this set. The data for test set A2 were collected similarly to the procedure for A1, as described above, but a different camera location was used. Set A2 was assembled following the same methodology as for sets A1 and B. For details, please see [14].

The database meets all of the requirements for the biometric assessment methodology. The correct classification (true acceptance) and false acceptance methodology were used in testing. For this purpose, each test set contained face images of people with (enrolled persons) and without (external persons, i.e., impostors) created face templates.

### 3.2. IFaViD’s Video Test Set

This section describes the size and parameters of IFaViD’s video test set containing video sequences and IFaViD’s image test set. The description of the size of the test is provided in Table 1. Scenario A represents human identification (1:N). For this reason, scenario A contains a relatively high number of impostors’ video sequences. Scenario B represents mainly human verification (1:1) or identification (1:N). Scenario B, therefore, contains a small number of impostors’ video sequences.

IFaViD’s image test set is presented in Table 2. The image test set contains face images only. The face images were extracted from IFaViD’s video sequences. Faces were detected by the Viola–Jones detector [16] with the face alignment framework.

To summarize, set A2 extends the database by approximately 62 thousand face images. This extension enlarges the database nearly four times, introduces a greater variability in the data, and enhances the overall credibility of the achieved results.

### 3.3. Evaluation Metrics

For the tests conducted on IFaViD, threshold-based metrics were used, as defined in Table 3. In this paper, we present results for both open-set and closed-set scenarios, aiming to provide a comprehensive and detailed analysis. In order to facilitate the orientation in the results, two other metrics are refined and added that help to distinguish between open- and closed-set identification. The correct classifications are used for both open- and closed-set tests and represent the fact that an enrolled individual is assigned the correct identity. False acceptance is used for open-set tests and enumerates how many impostors’ images are classified as any enrolled individual. The false classification is used for closed-set tests and enumerates images of enrolled individuals incorrectly assigned to a non-matching individual.

## 4. Application of TC Methods on the Data

This chapter describes and evaluates the application of TC methods to the data from the previous chapter. Firstly, we present the results of the cluster description-based methods. We compare the methods among themselves and identify the most suitable method for template creation, which will be used as a benchmark in the following tests. Secondly, we focus on the setup of the QIM method. Thirdly, we perform the evaluation of the cluster estimation-based methods. As test set A2 required a different setup of the QIM method compared to test sets A1 and B, we focus on an in-depth analysis and the results for test set A2 in the last part of this section. The achieved recognition performance of the proposed TC methods is presented in the form of ROC curves as an illustrative method commonly used in the field.

**Figure 3:**
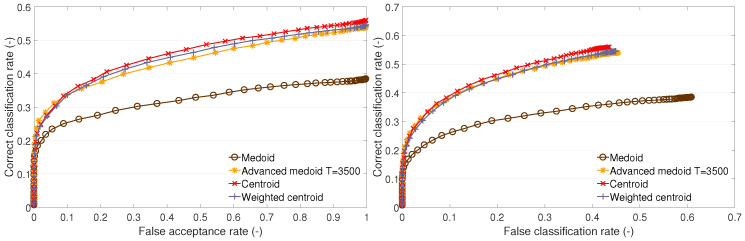
Recognition performance of cluster description—based methods on test set A1: ROC of open-set identification (**left**) and ROC of closed-set identification (**right**).

**Figure 4:**
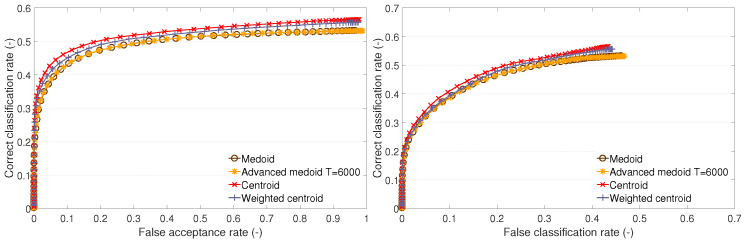
Recognition performance of cluster description—based methods on test set A2: ROC of open-set identification (**left**) and ROC of closed-set identification (**right**).

**Figure 5:**
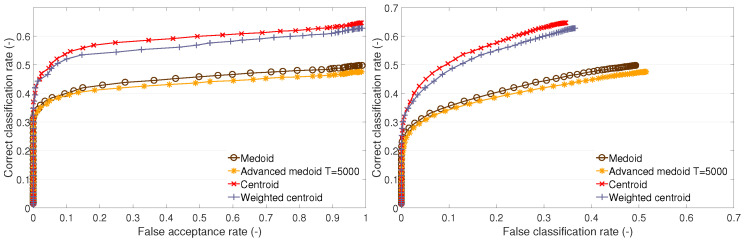
Recognition performance of cluster description—based methods on test set B1: ROC of open-set identification (**left**) and ROC of closed-set identification (**right**).

**Figure 7:**
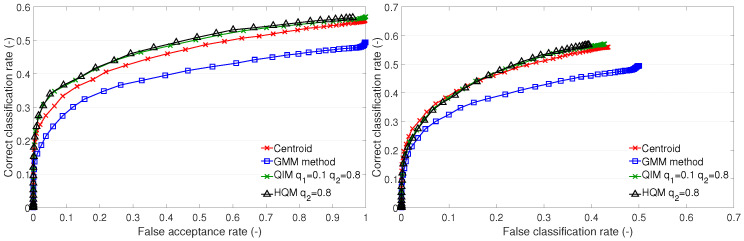
Recognition performance of cluster estimation-based methods on test set A1: ROC of open-set identification (**left**) and ROC of closed-set identification (**right**).

**Figure 8:**
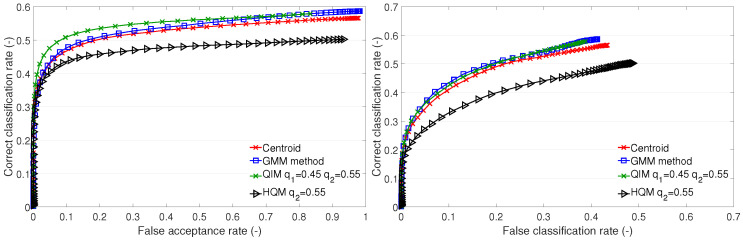
Recognition performance of cluster estimation-based methods on test set A2: ROC of open-set identification (**left**) and ROC of closed-set identification (**right**).

**Figure 9:**
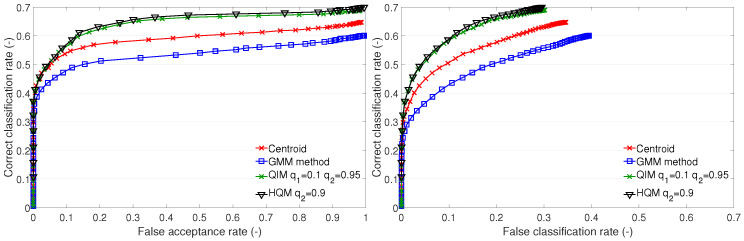
Recognition performance of cluster estimation-based methods on test set B1: ROC of open-set identification (**left**) and ROC of closed-set identification (**right**).

The cluster description-based methods and the GMM method were also used in our previous research [11,20]. In the subsequent sections (Section 4.1 and Section 4.3), we provide a comparison of the recognition performances of these methods via ROC curves (Figure 3, Figure 4 and Figure 5 and Figure 7, Figure 8 and Figure 9) on datasets A1, B, and the newly added A2. In addition, we distinguish between open- and closed-set identification. As demonstrated in these sections (Section 4.1 and Section 4.3), the QIM method outperforms all presented cluster description-based and cluster estimation-based methods, from which the centroid achieved the best results and, thus, was selected as the benchmark. (Further information can be found in [11,13,20]).

### 4.1. Evaluation of Cluster Description-Based Methods

This part presents the recognition performance of the following TC methods: the medoid method, the advanced medoid method, the centroid method, and the weighted centroid method. For each test set, the performance is presented on an open set (the dependency of the CC rate on the FA rate) and on a closed set (the dependency of the CC rate on the FC rate).

It can be observed that the traditional medoid method performs poorly on test sets A1 and B1 (Figure 3 and Figure 5) and performs similarly to the other methods on A2 (Figure 4). The advanced medoid method performs significantly better than the medoid on A1; on test set A2, it shows the same performance as the medoid. On B1 set, it outperforms the medoid, but the difference is not significant. The centroid shows the best performance on all of the test sets. It can be observed that the weighted centroid does not outperform the centroid; the performance is similar on A1 and A2, but, on B1, the weighted centroid fails compared to the centroid’s performance.

We give the following explanation of the test outcomes. The complexity of clusters formed by the training feature vectors is different. On training set A2, all methods perform almost with the same results. This suggests a low complexity of clusters, i.e., the clusters are compact. Neither the centroid, advanced medoid, nor weighted centroid indicates that the clusters’ representatives (templates) determined by these methods would be distant from the medoid because the performance is nearly the same. On training set A1, we can observe that the medoid fails compared to other methods. This implies that there can be outlying feature vectors that cause the medoid to fail. The other methods that either produce a synthetic template (centroid or weighted centroid) or enable outlying feature suppression (advanced medoid or weighted centroid) can cope with the nature of training data. Finally, training set B1 indicates highly complex training clusters because the methods producing synthetic templates (centroid or weighted centroid) significantly outperform the methods that determine one of the original training feature vectors (medoid or advanced medoid) as representative (template). The templates produced by the medoid and advanced medoid do not represent the clusters as well as the centroid or weighted centroid.

In addition to the recognition performance, we may also ask about the execution time. We can take into account the execution time of the TC methods (i.e., training stage) and the execution time during the recognition stage. As the training and template creation is carried out occasionally, there is no need to assess the execution time thoroughly. On the other hand, the execution time during the recognition stage is essential. The execution time is the same for all cluster description-based methods, which follows from the fact that the classifier performs an equal number of operations because the template vectors produced by all cluster description-based methods are of an equal length, i.e., dimensionality. Therefore, when comparing the templates produced by cluster description-based methods, the execution time is equal and does not play any role.

To summarize the results of the methods based on cluster description, the centroid method performs the best on all sets, and, considering its simplicity, it appears to be a suitable method for template creation. Therefore, it will be used as a benchmark in the following tests in which the cluster estimation-based methods presented below will challenge the centroid. In addition to investigating the performance of individual methods, the following finding is considered essential: the original training feature vectors do not appear to be optimal templates; synthetic representatives of a cluster perform the same or significantly better.

### 4.2. Setup of the Quantile Interval Method

**Figure 6:**
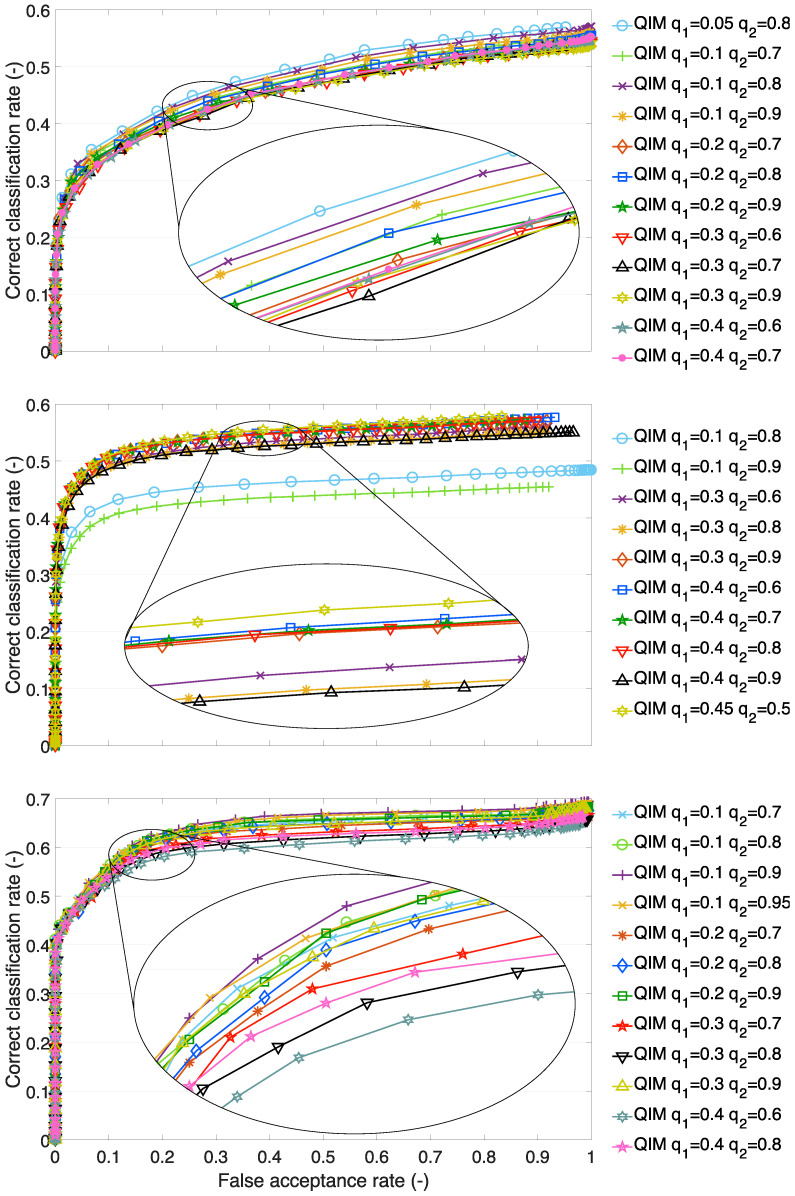
ROC of quantile interval method with different quantile values. Upper figure—test set A1, middle figure—test set A2, bottom figure—test set B1.

The setup of the QIM is given by selecting a PDF for the feature histogram representation and the quantiles. As a suitable PDF, the extreme value distribution was selected. The selection of quantiles was investigated in order to reveal how different quantiles influence the recognition performance. The results for different quantiles on test sets A1, A2, and B1 are shown in Figure 6.

In the case of test set A1, we can see that all quantile setups result in curves grouped together within the interval of ±3%. The setup $q_{1}=0.05$ and $q_{2}=0.8$ performs the best. As the lower quantile rises, and the upper quantile drops, the performance drops. On the A2 test set, we can observe grouped curves, too, except for two setups that cause performance drops. On the A2 test set, we can see the best performing setup with $q_{1}=0.45$ and $q_{2}=0.55$. A deep analysis of the quantile setup and the results for test set A2 are described further (see Section 4.4). The results conducted on test set B1 also show grouped curves, however, the differences in the performances among various setups are bigger, ±4%. The best-performing setup is for $q_{1}=0.1$ and $q_{2}=0.9$. Similarly to A1, as the lower quantile rises, and the upper quantile drops, the performance decreases.

### 4.3. Evaluation of Cluster Estimation-Based Methods

This section presents a discussion and evaluation of the performance of template creation methods based on cluster estimation, i.e., the GMM method, QIM, and HQM. For the comparison, the centroid method is also plotted.

The GMM method performs worse than the centroid on test sets A1 and B1 (Figure 7 and Figure 9). It performs similarly to the centroid on A2 (Figure 8). The GMM is an effective tool that allows for compartmentalization of data into multiple clusters. In this case, there is no more than one cluster per individual. Therefore, the setup of the GMM parameters, e.g., the number of components and the criterion of the best fit, should be determined with respect to representing one cluster. The optimal setup of the GMM parameters could improve the representation of the training features by the GMM and lead to better recognition performance. In the case of test set A2, it is expected that the training data form compact clusters, which are well represented by the GMM, resulting in a good performance.

Generally, the QIM performs best on all test sets. The presented results of the QIM have a different setup of quantiles for different test sets. For the A1 set, it is $q_{1}=0.1,q_{2}=0.8$; for A2, it is $q_{1}=0.45,q_{2}=0.55$; and, for B1, it is $q_{1}=0.05,q_{2}=0.95$. Generally, it appears that convenient quantile values are typical values for outlier detection, i.e., 0.05, 0.1, or 0.2. These setups perform well on test sets A1 and B1, with minor performance differences. The training set and test set A2 require different setups. The analysis of the QIM setup for A2 is included further (see Section 4.4).

The HQM performs well on test sets A1 and B1. This is due to a low value of the lower quantile used in the QIM, which gives values close to zero. In such cases, the optimization provided by the HQM is appropriate. In the case of test set A2, the QIM has a higher value of the lower quantile, and, therefore, the HQM fails. This allows for formation of assumptions for the usage of the HQM instead of the QIM. Firstly, the QIM template should be created, and the values of the lower quantiles should be investigated. Then, we should assess whether the HQM usage is appropriate. In the particular case of the LBPH features, the values of the features are natural numbers (non-negative integers). Therefore, it can be stated that we can use the HQM if the lower quantile of the QIM is $q_{1}\leq 0.1$. This assumption is fulfilled on subsets A1 and B1, and, thus, there is no performance difference between the HQM and QIM.

The QIM and HQM outperform the centroid method on test sets A1 and B1. On test set A2, the QIM outperforms the centroid too. The HQM fails on A2 for the above-mentioned reason. We may conclude that the QIM provides a better representation of the clusters than the centroid and the other methods. The drawback of the QIM (and also the HQM) is that it is a complex method dependent on the determination of a suitable PDF, PDF fitting, and setup of quantile values.

The execution time in the recognition stage is different for the selected cluster estimation-based methods. The templates produced by the GMM method, HQM, and centroid method have equal lengths, i.e., dimensionality. However, the template produced by the QIM contains two thresholds; therefore, the length is twice longer than the template produced by the other methods. Thus, the classifier has to carry out twice as many comparisons. This leads to a longer execution time, and, therefore, when using the templates produced by the QIM, the execution time should be taken into account.

### 4.4. Analysis of QIM Setup for Test Set A2

This part provides the analysis and explanation of why the QIM requires a different setup (a higher lower quantile and a lower higher quantile, i.e., narrow interval) for test set A2.

We have investigated the widths of the tolerance intervals for different setups of the QIM on different test sets. The selected widths for some setups are presented in Table 4, which shows a median of interval widths of all dimensions and all templates (all individuals) for the given set. The interval widths are similar for similar setups of the quantiles. However, the narrow tolerance interval performs best on test set A2. As the tolerance interval grows wider, the performance slowly drops, which has been shown in analysis of the dependencies of various quantile setups on the recognition performance in Figure 6. Wider intervals appear to cause misclassifications.

**Figure 10:**
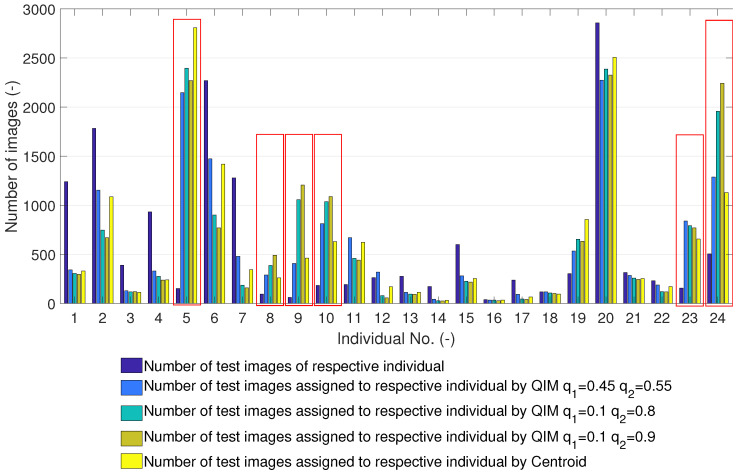
Misclassifications of enrolled individuals on test set A2 for different templates. Templates which are so generic that they are too similar to other faces are marked with red box.

To investigate the number and nature of incorrect classification, we worked out an overview of the classifier results, which is presented in Figure 10. The bar graph includes 24 groups of bars for each enrolled individual in the A2 set. For each individual, we depict a group of bars that show the actual number of test images of a particular individual and the number of test images assigned to the individual (template) by the classifier using differently created templates.

The bar graph (Figure 10) shows that there are some templates to which more test images than should be are assigned. In some cases, which are marked by a red box, the number of assigned test images is several times higher than the actual number of respective test images. This can be observed in the case of the QIM and centroid to slightly different extents. This results in the conclusion that these templates are too generic and cause misclassifications.

**Figure 11:**
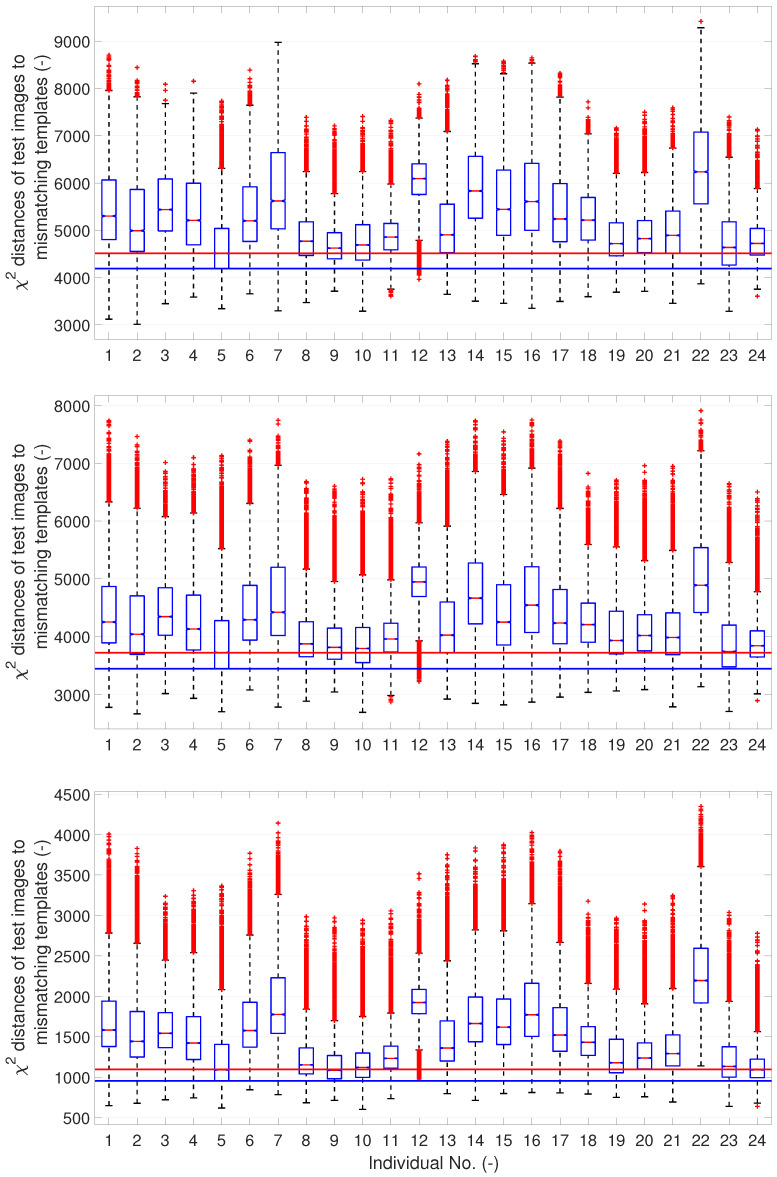
Distances of test images to mismatching templates in test set A2, Upper figure: for Centroid method, middle figure: for QIM $q_{1}=0.45$ and $q_{2}=0.55$, bottom figure: for QIM $q_{1}=0.1$ and $q_{2}=0.8$; the red line is the lowest median; the blue line is the lowest 25% quantile.

In order to confirm the nature of the templates, we evaluate the similarity of mismatching test images to templates on the A2 set. This evaluation should reveal whether some templates are more similar to the mismatching test images than others, which would explain why the templates marked by the red box in Figure 10 are assigned more test images than they should be. Figure 11 shows dissimilarities of the mismatching test images to the templates in the form of box plots; for details on box plots, see [27].

Figure 11 shows the dissimilarities among the mismatching test images and templates created by the centroid, the QIM $q_{1}=0.45$ and $q_{2}=0.55$, and the QIM $q_{1}=0.1$ and $q_{2}=0.8$. In the case of the centroid templates, we can observe a lower dissimilarity of template nos. 5, 9, 10, and 23. In the case of the QIM $q_{1}=0.45$ and $q_{2}=0.55$, we can observe a lower dissimilarity of template nos. 5, 8, 9, 10, 23, and 24. In the case of the QIM $q_{1}=0.1$ and $q_{2}=0.8$, we can observe low dissimilarities of templates nos. 5, 8, 9, 10, 19, 23, and 24. Moreover, the dissimilarities of template nos. 8, 9, 10, 19, and 24 are smaller (the box plots are lower) than in the case of the QIM $q_{1}=0.45$ and $q_{2}=0.55$. Generally, we can conclude that the templates of individual nos. 5, 9, 10, and 23 are too generic. This is caused by the training data, and none of the presented methods can produce more discriminative (less general) templates. There are some differences among the templates produced by different methods and setups, but none of the methods can substantially mitigate the generality of the training data.

The presented methods have not been designed to solve the issue of generic training data. They have been designed to: (i) choose optimal training data for creating templates from them, (ii) suppress the influence of unsuitable training data, and (iii) improve the representation of clusters. The issue of the training data and templates with low discriminative strength belongs to the area of discriminant analysis. However, the incorporation of discriminant analysis would require the creation of templates according to the variance of individual clusters. This approach has some drawbacks in application to surveillance and access control face recognition. Firstly, it requires the recreation of all templates when a new individual is to be enrolled. The quantile methods presented in this paper do not require this. Secondly, it introduces hardships to the template database management, which allows for disabling the templates when a particular individual is not present at the site.

To summarize the analysis of the setup of the QIM for test set A2, we can state that the extraordinary setup of the QIM for test set A2 is caused by the training data, which are too general. This results in templates that are too general and too similar to mismatching faces.

## 5. Achieved Results

Our contribution centers around face recognition and cluster analysis, specifically in the realm of template creation research. The primary objective of this research is to establish the most effective representation of the training data that optimizes recognition performance. We explore and investigate seven distinct template creation methods:

- The medoid method—uses the most similar training feature vector;
- The advanced medoid method—similar to the medoid method but rejects training vectors exceeding a certain dissimilarity threshold;
- The centroid method—the centroid of the training vector’s cluster is used;
- The weighted centroid method—the cluster centroid computation uses weights to decrease the importance of dissimilar training data;
- The GMM method—describes training data via the Gaussian mixture model. Then, the linear combination of the means and weights of the individual components is used;
- The QIM—represents a cluster as space, determined by the quantiles of the estimated distribution of the training data;
- The HQM—is an optimization of the QIM, which allows use of only higher quantiles for template construction.

**Figure 12:**
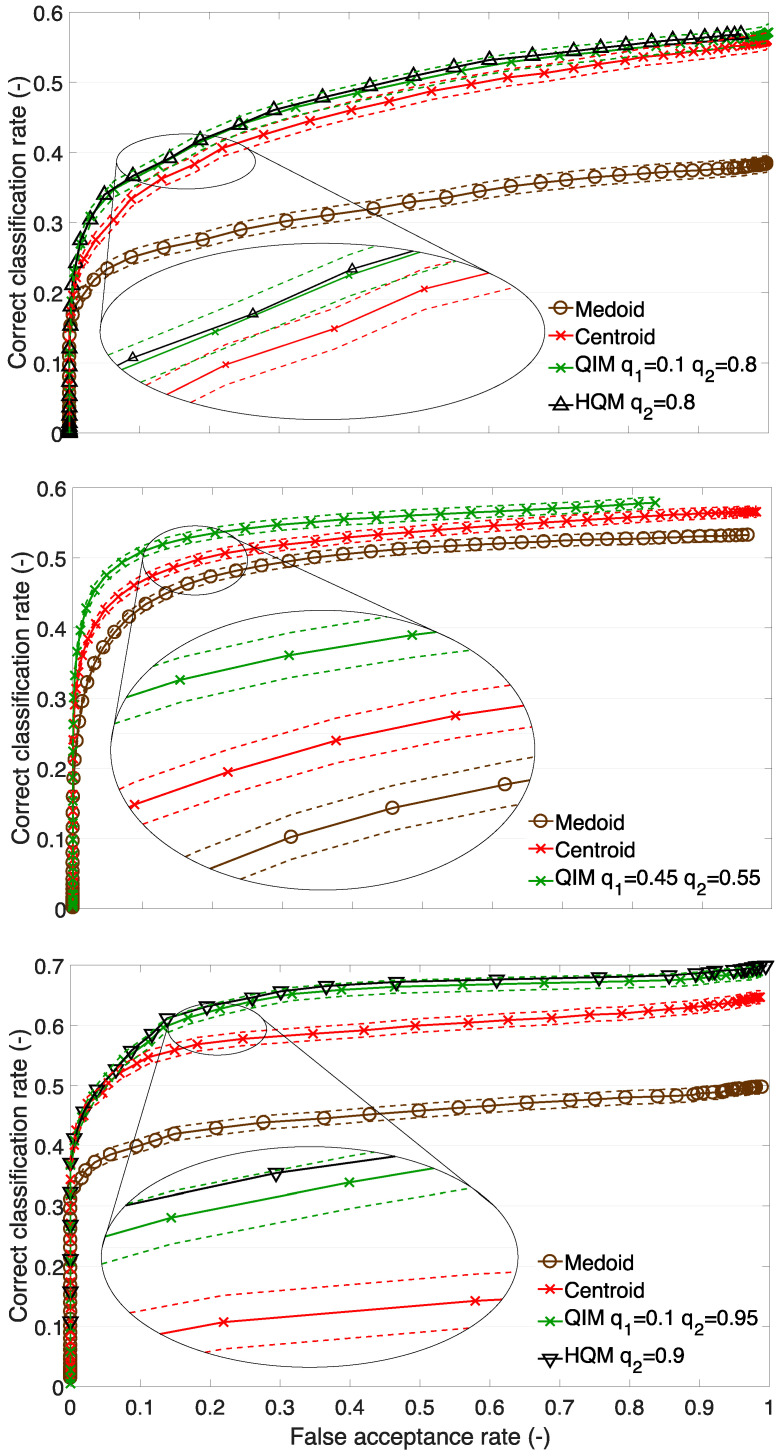
Recognition performance of selected methods. Upper figure—set A1, middle figure—set A2, bottom figure—set B1. Dashed lines show confidence intervals.

The methods listed above are used in this work to represent training face images in order to construct a face template. This is a pioneer work in the field of processing multiple training face images per person. Therefore, we began with the investigation of simple methods and made an effort to propose complex methods that would lead to further enhancement of the recognition performance. While some methods have certain drawbacks, we have focused on the most crucial ones for the purpose of this research and its documentation. These methods include the medoid, as a baseline; the centroid; the quantile interval method; and the higher quantile method. Figure 12 illustrates the outcomes of the mentioned template creation methods, thus consolidating all of the key findings in the area of template creation and cluster representation. A summary of the achieved improvement of the QIM method over the template creation methods based on cluster description and cluster estimation on scenarios A1, A2, and B1 in the percentage of CCR is presented in Table 5. Thus, the findings are:

1. Template creation and cluster representation are worth researching. It is shown that the proposed methods for template creation can enhance the recognition performance. The results can be observed in Figure 12 where the differences in the performance between the medoid method and quantile interval method are approximately 10–15% for test sets A1 and B1. This is considered the most important finding.
2. Feature vectors that represent a real face have proved not to be optimal cluster representatives. This is documented by the results of the medoid and advanced medoid methods. Synthetic representatives that do not correspond to a real face image enable a better recognition performance, i.e., the templates created by the centroid, weighted centroid, and other methods.
3. Simplicity is powerful—the centroid method provides templates that lead to a relatively high recognition performance. The centroid is a very simple and straightforward method that does not require any additional parameters.
4. The top performing and original method for template creation is the quantile interval method, which allows for an increase in the recognition performance by a range from 4 to 8% of the correct classification rate (against the centroid), see Table 5. The contribution of the QIM and HQM to the recognition performance is confirmed by the confidence intervals (see Figure 12). It is considered a newly established state-of-the-art method for face template creation. The HQM can speed up the classification process and decrease the memory requirements as its templates are half the length of the templates produced by the QIM.
5. If the data and the templates derived from it possess sufficient discriminatory characteristics, meaning they are not overly general, the QIM recognition method can be employed automatically. However, if the data and templates lack discriminatory ability, rendering them unsuitable for predefined and automated utilization of the method, manual setting of the quantiles becomes necessary. Under such circumstances, the method’s use is appropriate when the quantiles are set manually. Automating the method for cases where manual setting is required will be the focus of future research.

**Table 5:** The achieved improvement of correct classification rate (CCR) of the QIM.

QIM to:	Medoid	Advanced Medoid	Centroid	Weighted Centroid	HQM	GMM
A1	10–15%	3–4%	4–6%	3–5%	−1–0%	7–10%
A2	4–7%	6–9%	2–4%	4–7%	4–8%	2–7%
B1	10–15%	14–20%	4–9%	3–7%	−1–0%	6–11%

## 6. Conclusions

The paper focuses on exploring methods for creating robust and representative face templates in the face recognition process. Our primary objective is to improve and reinforce the evidence that the quantile interval method (presented in our previous work [11]) outperforms other contemporary face template approaches for template creation. To achieve this goal, we adopted the following approach.

Firstly, we expanded the image database used for testing, incorporating the IFaViD database. We introduced and used two scenarios representing surveillance applications and one scenario of an access control application. In addition, we distinguished between open- and closed-set identification. Secondly, we introduced and investigated six simple cluster description-based methods that are original in template research. Among these methods, we identified the centroid method as the best performer across all test sets. Due to its simplicity and effectiveness, the centroid method was selected as the benchmark against which we compared and challenged cluster estimation-based methods in further tests.

Thirdly, we presented the comparison of the contemporary approaches with the cluster estimation-based methods and proved that the QIM best outperforms the contemporary approaches. We further performed the QIM optimization, i.e., an analysis of the quantile settings. To reveal the influence of the quantile settings on the recognition performance, we explored both the lower and upper quantiles. The results obtained from test set A1 indicate that all quantile setups resulted in closely grouped curves within a ±3% interval. The results conducted on test set B1 also show the same result, however, the differences in performance among various setups are bigger, ±4%. In both cases, we observed that as the lower quantile increased, and the upper quantile decreased, the recognition performance declined. If the lower quantile of the QIM is $q_{1}\leq 0.1$, we recommend optimization of the QIM in the form of the HQM, i.e., the lower quantile is set to be zero. In the case of test set A2, our research revealed an improved performance for a narrower quantile interval (i.e., a higher lower quantile and lower higher quantile). As the tolerance interval grew wider, the performance slowly decreased. Therefore, we investigated the number and nature of the incorrect classifications. The results showed that some templates were assigned more test images than they should be, indicating that these templates were too generic, leading to misclassification. In order to confirm the nature of templates, we evaluate the similarity of mismatching test images to templates on the A2 set. The analysis reveals that some templates are more similar to the mismatching test images than others, which would explain why some templates are assigned more test images than they should be. We found out and proved that when the templates are too generic, wider intervals cause misclassifications, and, thus, we recommend a narrower quantile interval.

In our future work, we plan to focus on several directions. Firstly, we will continue researching the recognition performance by comparing the methods presented here with other approaches to face recognition. We will also assess the applicability of alternative methods in surveillance face recognition and access control systems. Secondly, our intention is to implement the described face recognition system in practical applications, starting with a server-based recognition engine. Subsequently, we aim to deploy the recognition engine to edge devices, such as surveillance cameras.

## Figures and Tables

**Table 1:** Description of the IFaViD’s video test set.

Scenario	A		B
Test set	A1	A2	B1
Number of: enrolled individuals/impostors	27/65	24/146	26/14
video sequences of enrolled individuals/impostors	619/223	742/1453	1082/41
Total number of Video sequences enrolled individuals/impostors	2443/1717		

**Table 2:** Description of the IFaViD’s image test set.

Scenario	A		B
Test Set	A1	A2	B1
Number of:			
enrolled individuals/impostors	27/65	24/146	26/14
images of enrolled individuals/impostors	6549/2 182	14,640/48,161	7342/369
Total number of:			
images enrolled individuals/impostors	28,531/48,753		

**Table 3:** IFaViD evaluation metrics.

Metric	Abbreviation	Description
	Enrolled individuals vs. impostors	
TA	True acceptance	Enrolled individual is classified to any enrolled individual
TR	True rejection	Impostor is classified as impostor
FA	False acceptance	Impostor is classified as enrolled individual
FR	False rejection	Enrolled individual is classified as impostor
	Enrolled individuals	
CC	Correct classification	Enrolled individual is correctly assigned
		matching identity
FC	False classification	Enrolled individual is incorrectly assigned
		non-matching
		identity

Note: It holds that CC+FC=TA.

**Table 4:** Tolerance interval widths for different QIM setups.

Quantiles of QIM		Median of Interval Widths for All Features and All Templates		
$q_{1}$	$q_{2}$	**A1**	**A2**	**B1**
0.1	0.8	1.34	1.21	1.18
0.1	0.9	1.67	1.40	1.37
0.45	0.55	N/A	0.13	N/A

## Data Availability

No new data were created or analyzed in this study. Data sharing is not applicable to this article.
